# Detection and biochemical characterization of insecticide resistance in field populations of Asian citrus psyllid in Guangdong of China

**DOI:** 10.1038/s41598-018-30674-5

**Published:** 2018-08-22

**Authors:** Fajun Tian, Xiufang Mo, Syed Arif Hussain Rizvi, Chaofeng Li, Xinnian Zeng

**Affiliations:** College of Agriculture, South China Agricultural University, Guangzhou, 510642 China

## Abstract

The Asian citrus psyllid, *Diaphorina citri* Kuwayama, is one of the most damaging pests of citrus-producing regions throughout the world. The use of insecticides is the main strategy for controlling psyllid and has increased year by year. In this study, four field populations of *D. citri* were evaluated for resistance to nine different insecticides using the leaf-dip method. The results showed that the highest level of resistance for *D. citri* was found in imidacloprid with a resistance ratio of 15.12 in the Zengcheng population compared with the laboratory susceptible population. This was followed by chlorpyriphos (6.47), dinotefuran (6.16), thiamethoxam (6.04), lambda-cyhalothrin (4.78), and bifenthrin (4.16). Piperonyl butoxide (PBO) and triphenyl phosphate (TPP) showed significant synergism on imidacloprid effects in the Zengcheng population (3.84- and 2.46-fold, respectively). Nevertheless, diethyl maleate (DEM) had no synergism on imidacloprid. Biochemical enzyme assays suggested that general esterase, glutathione *S*-transferase and cytochrome P450 monooxygenase activities were higher in the field-collected populations than in the laboratory susceptible population. However, glutathione *S*-transferase may play a minor role in the resistance of adult *D. citri* to insecticides. At the molecular level, resistance of *D. citri* to imidacloprid is mainly related to the increased expression of *CYP4C68* and *CYP4G70* (>5-fold).

## Introduction

The Asian citrus psyllid (ACP), *Diaphorina citri* Kuwayama, was first described in Taiwan in 1907, and now ACP can be found in most tropical and subtropical citrus-growing regions^1,2^. Both nymphs and adults of ACP suck leaf sap and produce large lots of honeydew, which ultimately causes a reduction in photosynthesis and growth of the fungus. In addition, ACP is a serious pest of citrus crops throughout the world because it is the vector of the bacteria causing huanglongbing (HLB)^3–6^. HLB symptoms are displayed on different parts of the plant; the fruits are under-developed, lopsided, and green colored with aborted or stained seeds, and the leaves are characterized by a yellow blotchy mottle or asymmetrical chlorosis^7^. Trees infected with pathogens eventually die within 5–10 years^8^. Because there is no available cure to treat diseased trees, management of HLB has focused primarily on the use of insecticides to control the vector to further reduce the spread of pathogens^9–11^.

At present, insecticides are a critical component of ACP management and are considered one of the most effective methods to manage the ACP. Insecticides with different modes of action (organophosphate, neonicotinoids, pyrethroids or carbamate) are often used to control the ACP. Eight to 12 treatments are commonly used each year in China, Florida, Brazil, Mexico and other major citrus-growing countries in the world^5,9^. Moreover, in some instances, growers repeated sequential use of the same insecticide or insecticides with the same mode of action. Under such selection pressure, the resistance of ACP to organophosphate, pyrethroid, neonicotinoid and carbamate insecticides has been observed. The first study on the susceptibility of different ACP populations was demonstrated in Florida in 2011^4^. Tiwari *et al*. found that the resistance ratio was up to 35.0-fold in one ACP population for imidacloprid, 17.9-fold for chlorpyriphos, 15.0-fold for thiamethoxam, 4.8-fold for fenpropathrin and 5.4-fold for malathion by using a topical application bioassay^4^. Subsequently, other studies suggested that ACP had different levels of resistance to insecticides in Arizona, California, Florida, India, Pakistan and Central West Mexico^1,6,12–18^. For example, Garcia *et al*. showed that ACP populations had become 106.5-fold and 4265.6-fold resistant to dimethoate and imidacloprid, respectively^19^.

The extensive use of insecticides has accelerated the selection pressure against insecticides. The ACP populations in different areas have varying levels of resistance to the same insecticide because the environmental conditions, inherent genetic differences between ACP populations, dosage of insecticide, insecticide application and exposures methods are not the same in different areas. Additionally, it is important to assess the severity of insecticide resistance in field populations of ACP to provide an early warning to change the chemical control strategies. However, there is still no study on the resistance level of ACP in field populations in different areas in China. Therefore, there is an urgent need to evaluate the severity of insecticide resistance to ACP populations in China and develop a resistance management strategy that maintains the effective use of all classes of insecticides for ACP control.

The high-level resistance in some insects was associated with an increase in general esterase activity or glutathione *S*-transferase or cytochrome P450 monooxygenase^20–22^. Previous studies have shown that insecticide resistance in ACP is correlated with detoxifying enzymes^4,15^. However, which of the three detoxifying enzymes played an important role was unknown. In general, pesticide bioassays, enzyme assays and the application of insecticide synergists are the basic procedures for acquiring preliminary information on potential resistance mechanisms. A typical characteristic of insecticide metabolic resistance is overexpression of detoxification genes at transcription level, resulting in increased protein amounts and enzyme activities that lead to higher level of detoxification and resistance development^23^. Tiwari *et al*. reported that five CYP4 genes (*CYP4C67*, *CYP4DA1*, *CYP4C68*, *CYP4DB1* and *CYP4G70*) were associated with the development of insecticide resistance in ACP^24^. Moreover, treatment with dsRNA caused reduced the expression of the five CYP4 genes and the mortality of ACP exposed to imidacloprid increased^25^. These results demonstrated that the five CYP4 genes were associated with imidacloprid resistance in ACP. However, it was unclear which of the five CYP4 genes played an important role in imidacloprid resistance.

The objective of this study is focused on the investigation of resistance levels in Guangdong populations of ACP to 9 commonly used insecticides. Baseline susceptibility data for ACP were determined by using a laboratory susceptible population. The synergistic effects of TPP, PBO and DEM were also evaluated in the most resistant and susceptible populations of ACP. Biochemical assays were carried out to determine the activities of detoxification enzyme, including general esterase, glutathione *S*-transferase and cytochrome P450 monooxygenase. To further investigate the resistance mechanism, the expression levels of the five CYP4 genes in the imidacloprid resistant and susceptible populations were compared to identify responsible candidate genes.

## Results

### Insecticide bioassay

The LC_50_ values, resistance ratios, 95% confidence limits and slopes for 9 insecticides for four field-collected populations of ACP are shown in Table 1. All field-collected populations showed different levels of resistance to nine insecticides. The Zengcheng population had the highest level of resistance to imidacloprid compared with the susceptible population, with an RR value of 15.12. The other populations displayed moderate levels of resistance to imidacloprid (Huizhou: RR = 5.48; Qingyuan: RR = 5.54; Conghua: RR = 6.62). One or more field populations showed moderate LC_50_ values to chlorpyriphos, thiamethoxam and dinotefuran. For example, the Zengcheng population displayed moderate level of resistance to dinotefuran (RR = 6.16). And the Huizhou population also had a moderate level of resistance to chlorpyriphos (RR = 6.47). For thiamethoxam, the Qingyuan population had a moderate level of resistance, and the resistance ratio was 6.04. There was no resistance to clothianidin, acetamiprid or chlorfenapyr.

**Table 1:** Log dose probit-mortality data for laboratory susceptible and four field populations of adult *Diaphorina citri* in response to different insecticides^a^.

Insecticide	Population	LC_50_^a^ (95% CL)^b^ (mg·L^−1^)	LC_95_ (95% CL)^b^ (mg·L^−1^)	Slope ± SE	χ^2^ (df)	RR_50_^c^	RR_95_^c^
Chlorpyriphos	Susceptible	1.48 bc (1.17–1.86)	6.24 b (4.38–10.95)	2.63 ± 0.35	0.91 (4)	—	—
	Zengcheng	2.33 b (1.80–3.05)	13.60 b (8.72–27.69)	2.15 ± 0.28	1.24 (4)	1.57	2.18
	Huizhou	9.58 a (7.20–13.80)	77.54 a (38.16–194.81)	1.92 ± 0.28	1.28 (4)	6.47	10.90
	Qingyuan	5.16 a (3.61–8.58)	63.60 a (27.82–302.79)	1.51 ± 0.25	0.87 (4)	3.49	10.19
	Conghua	1.16 c (0.86–1.56)	8.60 b (5.03–22.25)	1.89 ± 0.29	0.34 (4)	0.78	1.38
Bifenthrin	Susceptible	0.41 b (0.32–0.53)	2.21 c (1.48–4.06)	1.89 ± 0.23	3.38 (4)	—	—
	Zengcheng	0.59 b (0.43–0.78)	3.32 c (2.24–6.26)	2.20 ± 0.31	0.67 (4)	1.45	1.50
	Huizhou	0.59 b (0.41–0.81)	5.12 bc (3.12–11.58)	1.75 ± 0.25	1.84 (4)	1.44	2.31
	Qingyuan	1.70 a (1.17–2.52)	32.32 a (15.28–116.70)	1.29 ± 0.19	2.82 (4)	4.16	14.60
	Conghua	1.19 a (0.85–1.71)	12.90 ab (6.73–41.53)	1.59 ± 0.25	1.54 (4)	2.92	3.89
Lambda-cyhalothrin	Susceptible	0.70 c (0.50–1.08)	9.41 a (4.23–45.58)	1.46 ± 0.26	1.44 (4)	—	—
	Zengcheng	2.78 a (1.89–5.02)	37.03 a (14.66–254.58)	1.46 ± 0.27	1.26 (4)	3.96	3.94
	Huizhou	3.36 a (2.42–4.99)	37.82 a (18.99–123.89)	1.56 ± 0.23	3.65 (4)	4.78	4.02
	Qingyuan	1.59 b (1.15–2.18)	16.85 a (9.62–41.73)	1.60 ± 0.22	0.72 (4)	2.26	0.46
	Conghua	1.76 ab (1.26–2.49)	22.31 a (11.94–62.39)	1.49 ± 0.20	1.99 (4)	2.50	0.60
Imidacloprid	Susceptible	0.30 c (0.22–0.44)	3.31 b (1.70–10.49)	1.58 ± 0.23	1.72 (4)	—	—
	Zengcheng	4.57 a (3.37–6.87)	52.55 a (24.53–230.05)	1.55 ± 0.26	0.13 (4)	15.12	15.90
	Huizhou	1.65 b (1.18 2.33)	20.98 a (11.31–57.81)	1.49 ± 0.20	2.69 (4)	5.48	6.35
	Qingyuan	1.67 b (1.16–2.42)	26.79 a (13.47–85.37)	1.37 ± 0.19	1.48 (4)	5.54	8.10
	Conghua	2.00 b (1.43–2.91)	21.31 a (11.63–54.60)	1.60 ± 0.20	4.03 (4)	6.62	6.45
Thiamethoxam	Susceptible	0.55 c (0.36–0.97)	11.19 a (4.18–76.10)	1.25 ± 0.22	1.12 (4)	—	—
	Zengcheng	1.46 b (1.04–2.05)	18.13 a (9.98–47.95)	1.51 ± 0.20	1.43 (4)	2.69	1.62
	Huizhou	0.64 c (0.44–0.89)	7.03 a (4.22–15.69)	1.58 ± 0.21	3.69 (4)	1.17	0.63
	Qingyuan	3.29 a (2.47–4.59)	25.92 a (14.66–68.18)	1.84 ± 0.26	0.17 (4)	6.04	2.32
	Conghua	2.17 ab (1.54–3.13)	29.86 a (15.14–94.52)	1.44 ± 0.21	1.03 (4)	3.98	2.67
Clothianidin	Susceptible	0.39 a (0.26–0.66)	8.07 a (3.14–47.33)	1.25 ± 0.20	0.46 (4)	—	—
	Zengcheng	0.41 a (0.26–0.66)	11.42 a (4.92–44.69)	1.13 ± 0.15	1.36 (4)	1.05	1.41
	Huizhou	0.79 a (0.50–1.40)	25.30 a (9.55–130.67)	1.09 ± 0.15	1.89 (4)	2.05	3.13
	Qingyuan	0.57 a (0.36–0.98)	20.69 a (7.90–103.61)	1.05 ± 0.15	2.52 (4)	1.47	2.56
	Conghua	0.78 a (0.47–1.50)	41.35 a (13.08–310.30)	0.96 ± 0.14	1.49 (4)	2.03	5.12
Dinotefuran	Susceptible	0.37 c (0.25–0.63)	7.80 ab (3.05–45.05)	1.24 ± 0.20	1.44 (4)	—	—
	Zengcheng	2.28 a (1.61–3.35)	33.56 a (16.56–111.25)	1.41 ± 0.20	2.81 (4)	6.16	4.30
	Huizhou	1.51 ab (1.09–2.10)	17.11 ab (9.64–43.20)	1.56 ± 0.21	2.33 (4)	4.08	2.19
	Qingyuan	1.23 b (0.93–1.61)	7.98 b (5.22–15.39)	2.21 ± 0.26	1.56 (4)	3.31	1.02
	Conghua	0.42 c (0.28–0.46)	8.14 ab (3.81–28.21)	1.28 ± 0.17	2.48 (4)	1.13	1.04
Acetamiprid	Susceptible	2.16 ab (1.58–3.03)	22.63 a (12.43–61.01)	1.61 ± 0.22	2.85 (4)	—	—
	Zengcheng	2.67 ab (1.97–3.76)	25.70 a (14.14–69.34)	1.67 ± 0.23	3.39 (4)	1.26	1.14
	Huizhou	1.93 b (1.42–2.65)	18.15 a (10.46–44.47)	1.69 ± 0.23	2.15 (4)	0.89	0.80
	Qingyuan	2.27 ab (1.70–3.09)	18.19 a (10.76–42.43)	1.82 ± 0.24	4.73 (4)	1.05	0.80
	Conghua	3.98 a (2.93–5.83)	35.29 a (18.47–110.46)	1.74 ± 0.26	1.07 (4)	1.84	1.56
Chlorfenapyr	Susceptible	1.84 b (1.25–2.80)	40.81 a (18.17–168.26)	1.22 ± 0.18	1.56 (4)	—	—
	Zengcheng	2.67 ab (1.92–3.90)	33.45 a (16.90–107.70)	1.50 ± 0.22	2.88 (4)	1.45	0.82
	Huizhou	3.21 ab (2.31–4.76)	38.20 a (19.02–127.59)	1.53 ± 0.22	2.28 (4)	1.74	0.94
	Qingyuan	3.91 ab (2.73–6.24)	58.03 a (25.66–253.49)	1.40 ± 0.22	1.54 (4)	2.12	1.52
	Conghua	4.72 a (3.22–8.02)	75.43 a (31.03–394.12)	1.37 ± 0.22	2.23 (4)	2.56	1.85

LC_50_ and LC_95_ values followed by different letters within each group were significantly different from one another, based on non-overlapping of 95% confidence limit.^a^LC_50_ and LC_95_ values were measured by using 250–400 adults for each insecticide and population. ^b^CL = Confidence limit. ^c^RR (resistance ratios) = LC_50_ of field population/LC_50_ of susceptible population.

### Synergistic effects

As ACP had higher resistance to imidacloprid, the synergistic effects of TPP, PBO and DEM with imidacloprid were evaluated in Zengcheng-resistant and laboratory susceptible populations of ACP to measure the involvement of general esterase, mixed function oxidases and GST detoxifying enzymes in resistance mechanisms, respectively. The results are shown in Table 2. There was a significant difference between the LC_50_ values of the imidacloprid-PBO-treated and imidacloprid–treated Zengcheng populations. The toxicity of imidacloprid was nearly four times greater in the presence of PBO than in the absence of PBO. And the PBO also had the highest synergism in the susceptible population, but the synergistic ratio was only 1.67-fold. Meanwhile, a significant synergism was observed for TPP in the Zengcheng population (2.46-fold) but not in the susceptible population. For DEM, no synergy was found in the susceptible population and slight synergism was observed in the Zengcheng population. Therefore, the results showed that the resistance to imidacloprid was mainly caused by mixed function oxidases and general esterase detoxification.

**Table 2:** The synergistic effects of TPP, PBO and DEM on the toxicity of imidacloprid to the resistance and susceptible populations of *Diaphorina citri*^a^.

Population	Imidacloprid/Synergist	LC_50_^a^ (95% CL) ^b^ (mg·L^−1^)	Slope ± SE	χ^2^ (df)	RR^c^	SR^d^
Zengcheng	Imidacloprid	4.57 (3.37–6.87)	1.55 ± 0.26	0.13 (4)	15.12	—
	Imidacloprid + TPP	1.86 (1.31–2.70)	1.50 ± 0.20	0.78 (4)	6.20	2.46
	Imidacloprid + PBO	1.19 (0.81–1.76)	1.35 ± 0.17	0.74 (4)	3.97	3.84
	Imidacloprid + DEM	2.70 (1.80–3.95)	1.43 ± 0.20	1.56 (4)	8.67	1.69
Susceptible	Imidacloprid	0.30 (0.22–0.44)	1.58 ± 0.23	1.72 (4)	—	—
	Imidacloprid + TPP	0.24 (0.15–0.38)	1.15 ± 0.15	1.96 (4)	—	1.25
	Imidacloprid + PBO	0.18 (0.11–0.27)	1.25 ± 0.16	1.67 (4)	—	1.67
	Imidacloprid + DEM	0.27 (0.17–0.43)	1.18 ± 0.16	2.30 (4)	—	1.11

^a^LC_50_ values were determined by using 250-400 adults for each insecticide and population. ^b^CL = Confidence limit. ^c^RR (resistance ratios) = LC_50_ of field population/LC_50_ of susceptible population. ^d^SR (synergistic ratios) = LC_50_ without synergist/LC_50_ with synergist.

### Activities of detoxification enzymes

To further examine whether these detoxification enzymes were involved in insecticide resistance, we compared the general esterase, P450 monooxygenase and GST activities of the adults between field and susceptible populations of the ACP.

### Esterase activity

**Figure 1:**
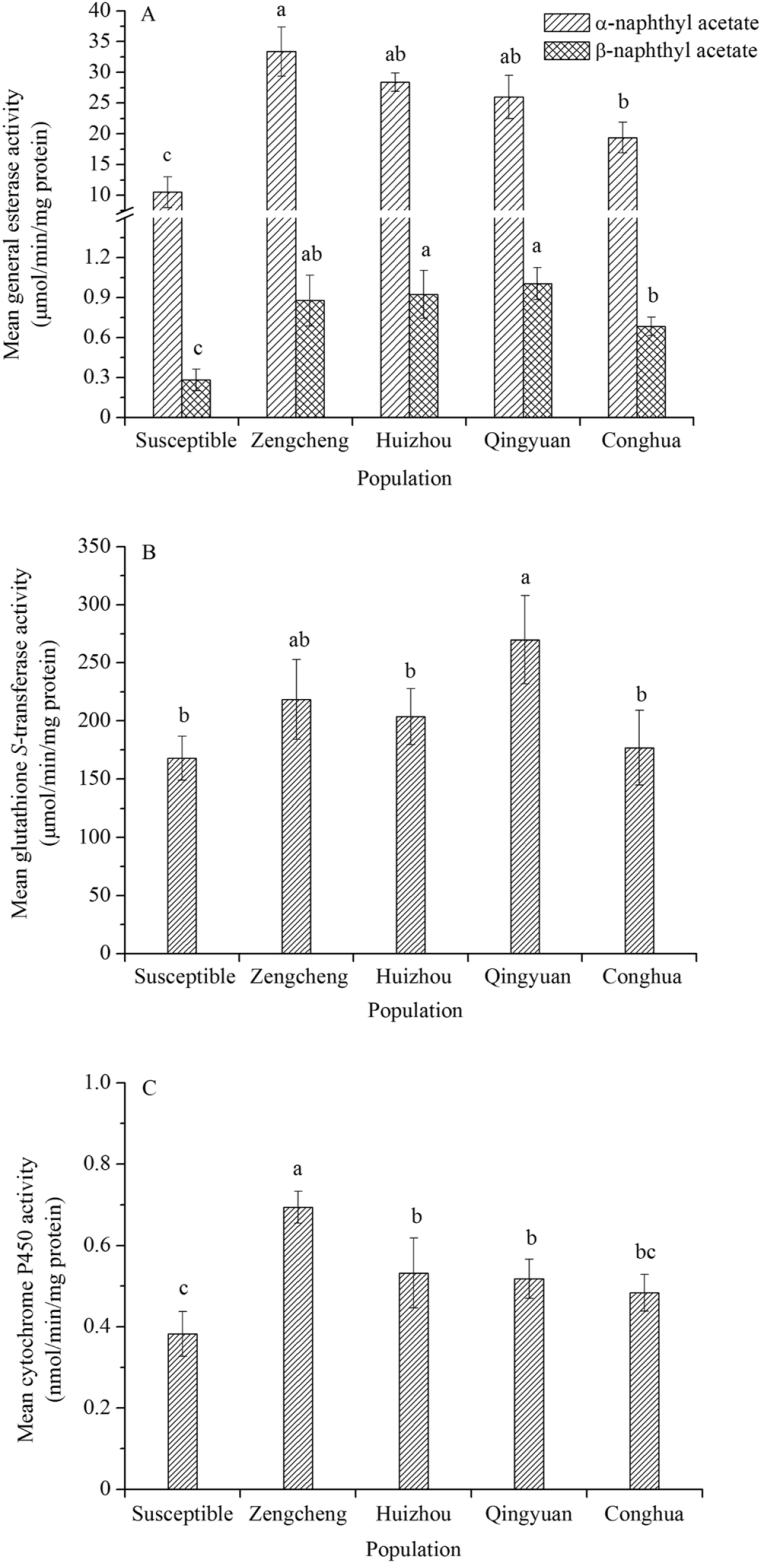
Comparison of (**A**) general esterase, (**B**) glutathione *S*-transferase and (**C**) monooxygenase levels between laboratory susceptible and four field populations of *Diaphorina citri* adults. The means within the same rank followed by different letters are significantly different at *p* < 0.05.

The results showed that the mean general esterase activity was significantly different among the populations (Fig. 1A). When α-NA was used as substrate, the general esterase activity of the Zengcheng and Huizhou populations was 3.18- and 2.71-fold higher than that of the susceptible population, respectively. However, the amount of general esterase was highest in the Huizhou and Qingyuan populations when β-NA was used as substrate. The general esterase activity was more than 3-fold higher than that in the susceptible population.

### GST activity

Mean GST activity was significantly higher in the population from Qingyuan than in the susceptible population (1.61-fold) (Fig. 1B). However, GST activity in the Zengcheng, Huizhou and Conghua populations was slightly higher than that of the susceptible population, and the difference was not statistically significant.

### P450 monooxygenase activity

The activity of P450 monooxygenase was measured with *p*-nitroanisole as a substrate. The results are shown in Fig. 1C. The activity was 2.09-, 1.60- and 1.56-fold higher in Zengcheng, Huizhou and Qingyuan populations than in the susceptible population. However, the Conghua population displayed lower activity that was not significantly different from the susceptible population.

### Five CYP4 genes expression

**Figure 2:**
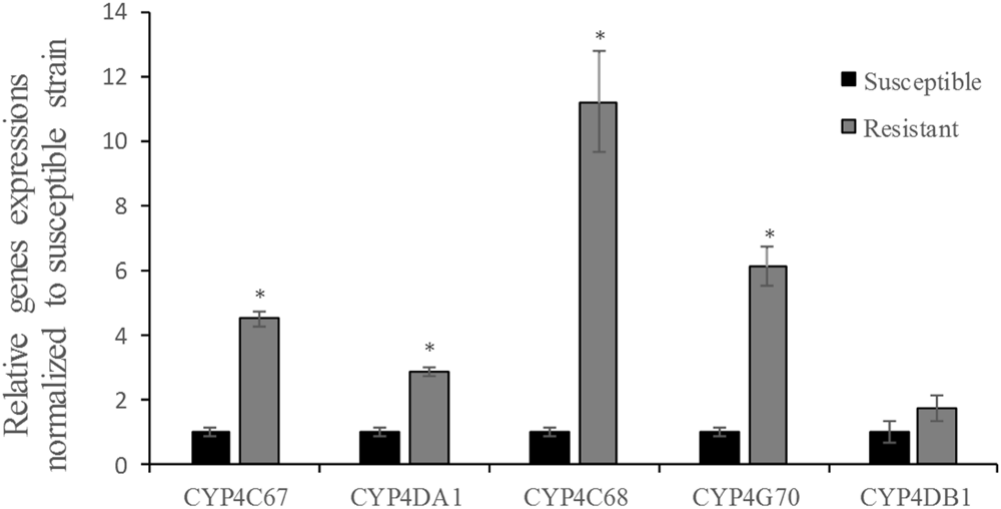
Expression profiles of five CYP4 genes in imidacloprid resistant and susceptible populations of *Diaphorina citri*. Relative gene expression was measured using qRT-PCR. C*t* values were first normalized to the reference gene, *β*-actin. Gene expression in the resistant *D. citri* population was normalized relative to gene expression in the susceptible *D. citri* population. Values are means ± SE of the three independent replicates. Asterisks indicates significant differences between the resistant and susceptible populations (Student’s *t*-test, *P* < 0.05).

**Figure 3:**
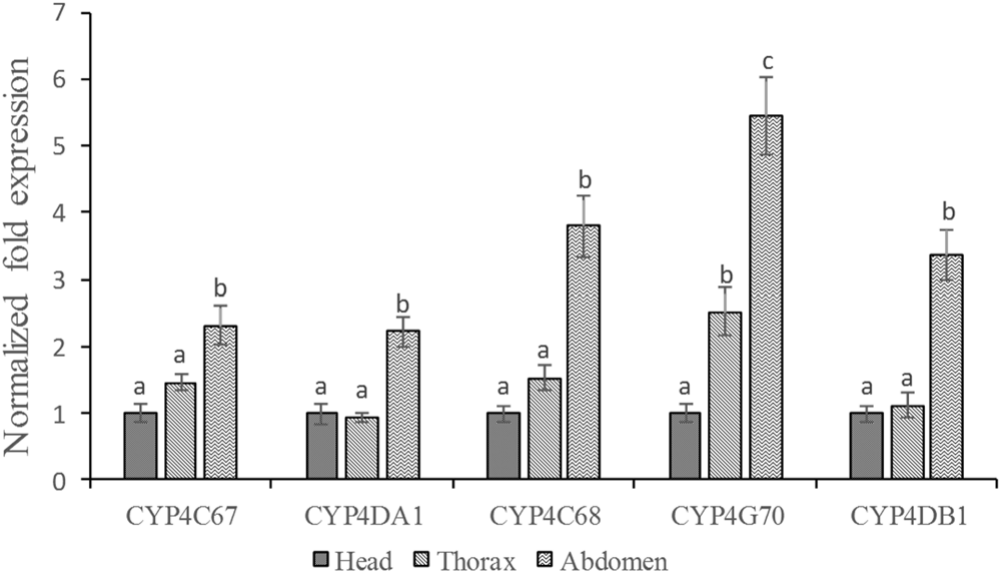
Expression patterns of five CYP4 genes in different tissues of the adult were evaluated by qRT-PCR. *β*-actin was used as an internal reference gene. The error bars represent the SE of the mean of the three independent replicates. Different letters on the bars indicate a significant difference among the different tissues (Tukey’s test, p < 0.05).

Based on qRT-PCR analysis, the expression levels of *CYP4C67*, *CYP4DA1*, *CYP4C68* and *CYP4G70* genes were significantly greater in the imidacloprid resistant population than in the susceptible population (Fig. 2). The relative expression of *CYP4C68* and *CYP4G70* was moderately high (>5-fold increase) in the imidacloprid resistant population. Moreover, the expression profiles of five CYP4 genes in different tissues (head, thorax and abdomen) of ACP adults (mature adult) were also examined by qRT-PCR. The expression levels were normalised with one reference gene, *β*-actin. The expression levels of the five CYP4 genes were significantly higher in the abdomen of the ACP (Fig. 3). For the *CYP4C67*, *CYP4DA1*, *CYP4C68* and *CYP4DB1*, there was no significant difference in the expression level between the head and thorax of ACP. In contrast, *CYP4G70* expression was also significantly higher in the thorax.

## Discussion

The ACP is an important pest and has been mainly controlled by the application of a wide variety of insecticides in China. Therefore, it is not surprising that many field populations have developed resistance to some insecticides. Monitoring populations under insecticide pressure played an important role in developing effective strategies for ACP control and resistance management. However, no data were available for the susceptibility of the ACP population to insecticides in China. In the present study, we tried to provide baseline susceptibility data of field populations of ACP to nine insecticides in Guangdong. The results showed that the LC_50_ data for the susceptible population were similar to those of other articles and thus it can be used as baseline susceptibility data^1,4^. The ACP had developed different levels of resistance to several insecticides among field populations when compared with the susceptible population. The highest level of resistance to imidacloprid was found in the Zengcheng population (RR = 15.12-fold) using the leaf-dip method. Similarly, a study by Tiwari *et al*. reported that the ACP had the highest level of resistance to imidacloprid in Florida via the topical application method (RR = 35.00-fold)^4^. It may be that the imidacloprid was often used to control ACP in Florida^5^. Moderate levels of resistance were detected in chlorpyriphos, thiamethoxam and dinotefuran. In contrast to this, the highest resistance ratios have already been reported for chlorpyriphos, thiamethoxam and bifenthrin in field populations of ACP^1,4^. There may be three factors associated with the resistance. First, frequent applications of chlorpyriphos, thiamethoxam and dinotefuran to control ACP on citrus^9^. Second, application of other insecticides promote cross-resistance with chlorpyriphos, thiamethoxam and dinotefuran^1^. Third, the enzymes general esterase, glutathione *S*-transferase and cytochrome P450 monooxygenase may contribute to the detoxification of chlorpyriphos, thiamethoxam and dinotefuran^4^. There was no resistance to clothianidin, acetamiprid or chlorfenapyr. Some studies also reported that ACP showed no resistance to clothianidin, acetamiprid and chlorfenapyr^3,4^. It may be that the use of insecticide mixtures or rotating different modes of action has allowed different populations of this insect to remain highly susceptible to clothianidin, acetamiprid and chlorfenapyr^1,16^. For example, Chen *et al*. have reported that the effective application of insecticides with various modes of action can delay or prevent development of resistance in populations of ACP. The susceptibility of ACP to dimethoate increased when the imidacloprid, fenpropathrin, abamectin + thiamethoxam, dimethoate and diflubenzuron are used in turn^16^. However, Naeem *et al*. suggested that ACP had a low level of resistance to chlorfenapyr in Punjab, Pakistan^1^. It may be that the resistance to chlorfenapyr was positively correlated with chlorpyrifos and bifenthrin, which showed very high levels resistance among the field populations of ACP in Punjab, Pakistan^1^. These factors may lead to the resistance differences of ACP to the same insecticide in different areas.

In general, synergistic research and enzyme assays are the significant steps to understand the detoxifying mechanisms of insecticide resistance. General esterase, GST and P450 monooxygenase metabolism have now been implicated in insecticide resistance in many insect species^20,26–28^. For example, the Colorado potato beetle *Leptinotarsa decemlineata’s* elevated P450 monooxygenase activity was involved in resistance to imidacloprid^26^. Synergists can be used to increase the toxicities of insecticides against insect pests by blocking metabolic enzymes of certain insecticides. When insecticide bioassays were carried out in the presence and absence of relatively specific synergists, the degree of synergism can indicate whether certain detoxification enzymes were involved in metabolic pesticides^29^. In the current study, as ACP had the most resistance to imidacloprid, the three synergists (TPP, DEM and PBO) were used to assess whether the toxicity of imidacloprid was enhanced to investigate the detoxifying mechanisms. The results showed that imidacloprid resistance in the Zengcheng population was significantly suppressed by TPP and PBO, suggesting a possible involvement of both general esterase and P450 monooxygenase in imidacloprid resistance (Table 2). Meanwhile, the activities of general esterase and P450 monooxygenase in the Zengcheng population increased 3.18- and 2.09-fold, respectively (Fig. 1). This was in accordance with the present synergism results and those of previous studies^13,15^. Therefore, the bioassay and biochemical results indicated that general esterase and P450 monooxygenase played important roles in imidacloprid resistance in the Zengcheng population. Many studies have also shown that increased activities of general esterase and P450 monooxygenase are common insecticide resistance mechanisms against organophosphates, pyrethroid and neonicotinoid insecticides in some insect pests^20,30,31^. From the results of enzyme activity, we also concluded that general esterase and P450 monooxygenase were important factors in resistance of ACP to insecticides.

GST was also responsible for metabolizing xenobiotic compounds as well as endogenous compounds. It has been reported that the resistance of some insect pests to insecticides is associated with enhanced GST activity^32^. The results from the present study suggested that the mean GST activity in the Qingyuan population of ACP was 1.61-fold higher than in the susceptible population (Fig. 1B). However, the GST activity in other field populations was slightly higher than that of the susceptible population and was not statistically significant. Meanwhile, no synergy was found for DEM in the susceptible population, and slight synergy was observed in the Zengcheng population (1.11- and 1.69-fold, respectively). Therefore, GST does not seem to have a significant effect on resistance to imidacloprid. And GST also plays a minor role in the resistance of the ACP to insecticides.

Previous studies suggested that the five CYP4 genes were associated with imidacloprid resistance in ACP^24,25^. However, which of the five CYP4 genes played an important role was unknown. Therefore, we compared transcript expression of the five CYP4 genes in imidacloprid resistant and susceptible populations. Four CYP4 genes (*CYP4C67*, *CYP4DA1*, *CYP4C68* and *CYP4G70*) were overexpressed in the imidacloprid resistant population, and overexpression was highest for *CYP4C68* and *CYP4G70* (>5-fold) (Fig. 2). Overexpression of CYP4 genes involved insecticide resistance has been reported in many insect species^33,34^. For example, resistance to imidacloprid in field populations of *Bemisia tabaci* was associated with the increased expression of *CYP4C64* gene^33^. Therefore, these results suggested that imidacloprid resistance in ACP was mainly related to overexpression of *CYP4C68* and *CYP4G70*. However, the reduced penetration and target-site modifications within detoxifying enzymes may also play an important role in resistance development among the field-collected populations of ACP. A study by Liu *et al*. reported that the resistance of brown planthopper to imidacloprid was related to the target-site modifications and enhancement of detoxification. The identified target-site mutations, Y151S, within two nAChR subunits (Nlα1 and Nlα3) were involved in imidacloprid resistance^35^. Therefore, in the next step, we will focus on the transcriptome and analyze the detoxification enzyme gene to require a more comprehensive understanding of the metabolic resistance of ACP to imidacloprid.

To investigate the expression profiles of the five CYP4 genes, the different tissues of the adult were analyzed by using qRT-PCR. The five CYP4 genes were found most abundant in the abdomen (Fig. 3). Imidacloprid is a stomach toxicant, indicating that higher expressions of five CYP4 genes in abdomen may be related to how the tissue encounter insecticides. Midgut is usually considered to be the major detoxification organ in insects^36^. The midgut presented in the abdomen of the insect. Therefore, the result of tissue-dependent expression patterns are consistent with this notion for the five CYP4 genes. However, imidacloprid is also a contact toxicant and can be absorbed by epidermis of insect through contact with residues on crop surfaces. So the next step we will investigate the expression profiles of the five CYP4 genes in the epidermis. It help us better understand the resistance mechanism of ACP to imidacloprid.

In China, the application of different insecticides to control the ACP has a long history. No data were available for monitoring insecticide susceptibility in field populations of ACP in China in different years. Insecticide resistance management should be carried out before the discovery of product failures in the field. In this study, we determined baseline susceptibility data of field populations of ACP to commonly used insecticides by the leaf-dip method. The resistance mechanism of ACP was to enhance the activities of general esterase and P450 monooxygenase. And four CYP4 genes (*CYP4C67*, *CYP4DA1*, *CYP4C68* and *CYP4G70*) were overexpressed in the imidacloprid resistant population, and overexpression was highest for *CYP4C68* and *CYP4G70* (>5-fold). However, our finding don’t exclude the possibility that other multiple detoxification genes and target-site are involved in ACP resistance to imidacloprid. Therefore, these resistance mechanisms need further study. This may help to gain a better understanding of other resistance mechanisms in ACP and develop some strategies to avoid the development of resistance. Moreover, continued resistance monitoring of ACP is needed to record whether resistance levels will continue to increase, and this situation may persist for several years due to extensive use of insecticides for ACP management. Therefore, a resistance management strategy could be designed and performed by rotating insecticides with different modes of action, pheromone traps and incorporation of new modes of action to extend the efficacy of currently available insecticides against ACP.

## Materials and Methods

### Insects

The laboratory susceptible population of ACP was continuously reared at the Guangdong Engineering Research Center for Insect Behavior Regulation, South China Agricultural University, Guangdong, China. The culture was established in 2008 prior to the discovery of HLB. This population was maintained on sweet orange (*Citrus sinensis* (L.)) seedlings without exposure to insecticides in a greenhouse at 26 ± 2 °C, with 60 ± 5% relative humidity (RH) and a 14/10 light/dark photoperiod. Adult ACP populations were collected by mouth aspiration from four commercial citrus groves in Guangdong, China, from the following regions: Zengcheng (23°23′47′′N, 113°49′6′′E), Huizhou (23°19′58′′N, 114°7′52′′E), Qingyuan (23°38′53′′N, 112°49′36′′E) and Conghua (23°46′28′′N, 113°49′59′′E). These places were chosen for heavy insecticide usage in the citrus groves for the management of ACP and several other insect pests. After collection, they were transported to the laboratory and fed on citrus plants in Plexiglas cages (50 × 50 × 50 cm) until use for bioassays. An imidacloprid resistant population of ACP was developed from a field population which was collected from Guangzhou in Guangdong Province, south China, and then they were selected with exposure to imidacloprid. Imidacloprid selection was performed at every generation by treating 1000–1500 adults (mixed sex) topically with the LC_50_ of that generation (average mortality 50–60%). After 9 generations of continuous selection, the population showed 52.19-fold resistance to imidacloprid by leaf-dip method in the ACP adults. The resistant population was also maintained in the laboratory under the conditions described above.

### Insecticide and reagents

The insecticides used in all bioassays were analytical grade. They included chlorpyriphos (99.2%), bifenthrin (99.0%), lambda-cyhalothrin (97.5%), imidacloprid (98.3%), thiamethoxam (99.3%), clothianidin (98.6%), dinotefuran (99.1%), acetamiprid (99.6%), and chlorfenapyr (98.9%) belonging to different chemistry classes and modes of action. Insecticides were purchased from Qinchengyixin Technology Development Co., Ltd. (Beijing, China). Synergist piperonyl butoxide (PBO), triphenyl phosphate (TPP) and diethyl maleate (DEM) were obtained from Sigma Chemical Co. (St. Louis, MO) at the highest purity available. The other chemicals were purchased from commercial suppliers. They were stored in accordance with the manufacturer’s instructions.

### Toxicity bioassay

In the present study, the leaf-dip method was used for assessing direct toxicity to different insecticides^15,37^. For the bioassay, 3 mL of 1.5% agar solution was added to 60-mm-diameter plastic disposable petri dishes (Jiangsu Datang Medical Equipment Co., Ltd, Jiangsu, China) to form a solidified bed. All the fresh leaves of sweet orange (*Citrus sinensis* (L.)) without exposure to insecticides were collected from the green house in our campus, which are firm, fully expended and dark green. Leaf disks (60 mm diameter) were excised and treated with insecticide-acetone solutions for 30 s and then left in a fume hood at room temperature for an hour for drying. Leaf disks treated with acetone were used as a control treatment. ACPs were briefly anesthetized with a short puff of CO_2_ to facilitate handing and transfer. After one hour, leaf disks were placed on agar beds, and ten adult ACPs were transferred into each dish using a soft camel-hair brush. The parafilm was stretched and placed on the top of the petri dishes to prevent escape of adults. Five or six concentrations of each insecticide were tested. Each concentration of an insecticide was replicated 3 times, and each experiment was repeated twice. Mortality was assessed after the treated adults were held in a growth chamber at 25 ± 1 °C, 60 ± 5% RH and 14/10 light/dark photoperiod for 24 h. The criterion for death was that an adult failed to make a coordinated movement when probed with a camel hair brush.

### Synergism test

TPP, DEM and PBO affected general esterase, glutathione *S*-transferase and mixed-function oxidase detoxifying enzyme activities, respectively. To determine the synergistic effects, we performed a bioassay pre-experiment with different concentrations of synergists to select the appropriate dose of synergists. The highest concentration of each synergist that could cause comparable mortality with the control (acetone) was chosen for the final bioassay^38^. The different concentrations of insecticides were mixed with an equal volume of 20 mg·L^−1^ synergist. The final concentrations of PBO, TPP and DEM were 10 mg·L^−1^. The bioassays were performed using the leaf-dip method described in the toxicity bioassay section. Meanwhile, the experimental conditions and the criterion for death were the same as described above.

### Enzyme preparation

Fifty adults were homogenized using a handheld homogenizer with a plastic pestle in 300 μL of ice-cold phosphate buffer (0.1 M, pH 7.5, containing 0.1% Triton X-100) in a 1.5 mL centrifuge tube. The homogenates were centrifuged for 15 min at 10,000 *g* at 4 °C. Thereafter, the resulting supernatant was transferred into a 1.5 mL single-use centrifuge tube. Next, the supernatant was diluted appropriately by the addition of homogenization buffer without Triton X-100. It was placed on ice and served as the enzyme source in the general esterase and glutathione *S*-transferase reaction mixtures.

For the P450 monooxygenase activity assays, 200 adults were homogenized on ice in 1 mL sodium phosphate buffer (0.1 M, pH 7.8) containing 1 mM EDTA, 1 mM dithiothreitol (DTT), 1 mM propylthiouracil (PTU), and 1 mM phenylmethane sulfonyl fluoride (PMSF). The homogenate was centrifuged at 10, 000 *g* for 30 min at 4 °C. The supernatant was carefully transferred into a 1.5 mL centrifuge tube to ensure that no debris was carried and immediately placed on ice for monooxygenase assay^39^. The protein content for each enzyme source sample was measured by the Bradford method using bovine serum albumin (BSA) as the standard^40^. The absorbance was determined in a 96-well microplate reader at 595 nm after the mixture was incubated for 5 min at 25 °C.

### General esterase assay

α-Naphthyl acetate (α-NA) and β-naphthyl acetate (β-NA) were used as substances to determine general esterase activity^41–43^. The reaction mixture in each microplate well contained 15 μL of enzyme solution and 135 μL of 0.3 mM α-NA or β-NA. Next, 50 μL of fast blue B-salt in 5% SDS solution was added to the reaction mixture after incubating the mixtures at 37 °C for 30 min. After another incubation period at room temperature for 15 min, the activity of general esterase was determined at 595 nm at 25 °C. 15 μL of 0.1 M phosphate buffer substituted enzyme solution was used as the control. The activity of general esterase was reported as nanomoles of product formed/min/mg protein using α-naphthyl and β-naphthyl standards for α-NA and β-NA, respectively.

### Glutathione *S*-transferase (GST) assay

The activity of GST was determined using 1-chloro-2,4-dinitrobenzene (CDNB) as the substrate^44–46^. The reaction mixture in each microplate well contained 10 μL of enzyme solution and 190 μL of CDNB-GSH solution [10.35 mM GSH in phosphate buffer (0.1 M, pH 7.5) and 200 mM CDNB in 188:2 (v/v) ratio]. To determine the activity of GST, the absorbance was measured kinetically for 5 min at 340 nm at 25 °C. Ten microliters of phosphate buffer (0.1 M, pH 7.5) as a substitute for the enzyme solution was regarded as the control. Four replicates were carried out for each population of ACP. The extinction coefficient of CDNB (9.6 mM^−1^·cm^−1^) was applied to correct the path length of the solution in the microtitre plate well, and the activity of GST was determined as micromoles of conjugate produced/min/mg protein.

### Cytochrome P450 monooxygenase assay

Cytochrome P450 activity was quantified using *p*-nitroanisole as a substrate^32,39,47^. Then, 100 μL of 2 mM *p*-nitroanisole solution, 10 μL of 9.6 mM NADPH and 90 μL of enzyme stock solution were added to each well a microplate and mixed. The plate was incubated for 30 min at 34 °C in an air atmosphere. The absorbance was determined at 405 nm at 30 °C. The activity of cytochrome P450 was expressed as nanomoles of *p*-nitrophenol/min/mg protein using the standard curve of *p*-nitrophenol.

### RNA isolation and cDNA synthesis

Total RNA was extracted using RNA extraction kit (TaKaRa, Japan) based on the manufacturer’s introductions. And the total RNA of different tissues (head, thorax and abdomen) of ACP adults (mature adult) were also extracted using the same method as described above. The resulting total RNA was resuspended in nuclease-free water and quantified with the spectrophotometer (Thermo Scientific Nanodrop 2000). Then, cDNA was synthesized with the PrimeScript^TM^ RT reagent Kit with gDNA Eraser (Takara Biotechnology, Dalian, China) according to the manufacturer’s introductions.

### Quantitative real-time PCR

The genome of ACP possesses five CYP4 genes; *CYP4C67* (JF934716), *CYP4C68* (JF934717), *CYP4DA1* (JF934718), *CYP4DB1* (JF934719) and *CYP4G70* (JF934720). They were identified by Tiwari *et al*. and associated with the development of insecticide resistance in ACP^24^. A total 90 adults (three biological replicates, n = 30) of imidacloprid resistant and susceptible populations were subjected to qRT-PCR analysis. Primers were listed in Table 3. The qRT-PCR was carried out in a 20 μL reaction volume containing 10 μL of Go Taq® qRCR Master Mix, 1 μL of each primer, 1 μL of diluted cDNA and 7 μL of nuclease-free water (Promega, Madison, WI, USA). Real-time PCR was performed using the following conditions: 95 °C for 2 min, followed by 40 cycles of 95 °C for 15 s, 59 °C for 30 s. The final melting-curve were determined by taking continuous fluorescence readings whilst increasing the temperature from 59 °C to 95 °C in 0.5 °C increments, keeping 30 s after each 0.5 °C increment. PCR specificity was examined by melting curve analysis and by verification of single amplicons using on agarose gels. The relative expression levels for the target genes were calculated by the 2^−∆∆*C*t^ method^48^. The expression levels were normalised with one reference gene *β*-actin.

**Table 3:** Sequences for quantitative real-time PCR (qRT-PCR) primers.

Gene	Forward Primer sequence (5′ to 3′)	Reverse Primer sequence (5′ to 3′)
CYP4C67	TGGAACGTGTCATCAAGGAG	CCGGATTGAAACTGTTAGGC
CYP4DA1	AGTGGTGTCGGAAATTGAGG	GTTCGAGCCACCTGGAGATA
CYP4C68	CTAGCCTGGACCCTCTTCCT	ACCCTCCCTATGAACGGAAC
CYP4G70	GCCGGAAGTTCTTTCTTCCT	TAACGGGTACTGGTGGGAAC
CYP4DB1	CTGTACGCTCTGGGACATCA	TTGAGCGGTGCATAGAGTTG
*β*-Actin	TGTGACGAAGAAGTTGCTGC	TGGGGTATTTCAGGGTCAGG

### Statistical Analysis

The bioassay data were subjected to probit analysis using the POLO program PC PoloPlus (LeOra Software, Berkeley, CA)^49^. Resistance ratio (RR) were determined as the LC_50_ of the field population/ LC_50_ of the susceptible population. The level of resistance was selected from the previous reported resistance in ACP against the same classes of insecticide^4,46^. Synergistic ratio (SR) were calculated as the LC_50_ without synergist/LC_50_ with synergist. Statistical analysis was carried out by one-way ANOVA on SAS (version 9.2), and comparisons of the means were made by Fisher’s LSD test to determine differences in detoxifying enzymes between susceptible and field-collected populations. Statistical significance was evaluated using one-way ANOVA (SPSS; version 19.0 for Windows) followed by the Tukey’s test at the 0.05 level.
